# Diagnosing Myocardial Contusion after Blunt Chest Trauma

**Published:** 2016-04-13

**Authors:** Zahra Alborzi, Vahid Zangouri, Shahram Paydar, Zahra Ghahramani, Masih Shafa, Bizhan Ziaeian, Mohammad Reza Radpey, Armin Amirian, Shahin Khodaei

**Affiliations:** 1 *Department of Cardiology, Medical School, Yasouj University of Medical Sciences, Yasouj, Iran.*; 2 *Department of General Surgery, Shiraz University of Medical Sciences, Shiraz, Iran.*; 3 *Trauma Research Center, Shahid Rajaee (Emtiaz) Trauma Hospital, Shiraz University of Medical Sciences, Shiraz, Iran.*; 4 *Department of Cardiac Surgery, Shiraz University of Medical Sciences, Shiraz, Iran.*; 5 *Department of Thoracic Surgery, Shiraz Universityof Medical Sciences, Shiraz, Iran.*; 6 *Department of Vascular Surgery, Shiraz University of Medical Sciences, Shiraz, Iran.*

**Keywords:** *Thoracic injuries*, *Contusion*, *Diagnose*

## Abstract

A myocardial contusion refers to a bruise of the cardiac muscle, the severity of which can vary depending on the severity of the injury and when the injury occurs. It is a major cause of rapid death which happens after blunt chest trauma and should be suspected at triage in the emergency department. We demonstrated that suspected myocardial contusion patients who have normal electrocardiograms (ECGs) and biomarker tests can be safely discharged. However, if the test results are abnormal, the next steps should be echocardiography and more advanced measures. Diagnosing myocardial contusion is very difficult because of its nonspecific symptoms. If a myocardial contusion happens, cardiogenic shock or arrhythmia must be anticipated, and the patient must be carefully monitored.

## Introduction

Myocardial contusion is an uncommon complication of blunt chest trauma; it is more likely to occur from injuries resulting from accidents related to a sudden decrease in vehicle speed.^1^ Diagnosing myocardial contusion is very difficult due to the patient’s nonspecific symptoms and the lack of an ideal diagnostic test. Several methods are available for its detection, such as Electrocardiography (ECG), echocardiography, nuclear cardiac imaging, and heart biomarkers, none of which have 100% sensitivity.^2^^, ^^3^ Phase approach to patients with suspected myocardial contusion will lead to a significant reduction in the number of trauma patients admitted to the hospital and a reduction in the number of unnecessary diagnostic procedures.^4^ It has been documented that implementing ECG in the first stage of treating a person with a traumatic contusion (significant thoracic trauma) on the chest shows if the probability of contusion is high; thus, it is a class I recommended diagnostic test.^5^ Significant thoracic trauma could include multiple rib fractures, simultaneous pulmonary contusion, and hemothorax.

In these conditions, one is strongly recommended against adding biomarkers to the ECG. In cases of an abnormal ECG, however, measuring biomarkers at the beginning of hospitalization and again 4-6 hours later will increase the negative predictive value of this method alone. Measuring biomarkers is part of the predictive negative value of class II.^5^^, ^^6^ If the results of tests in steps 1 and 2 are abnormal, or if the patient is in shock and cardiac causes are being considered, an echo or long-term monitoring (24–48 hours) is recommended.^7^^-^^10^ The most recent studies have minimized the necessity of echocardiography in sternal fractures.^1^ Therefore, a recommendation for routine echocardiography is of limited value.

Diagnosing myocardial contusion in trauma patients in the emergency department is still challenging, and the most advanced diagnostic tests are expensive and unavailable under the current dire conditions in Iran. This study attempted to provide the appropriate diagnostic algorithm and the steps to eliminating many of the uncertainties in dealing with such patients. 

***Statement of Problem***

Based on the prevalence of trauma in urban communities and the lack of specific tests for the diagnosis of contusion and myocardial contusion, we decided to suggest a step-by-step diagnostic logic as a guideline which would reduce unnecessary diagnostic costs and long-term hospitalizations for trauma patients. We should devise a safe protocol that is appropriate for managing patients with suspected myocardial contusions referring to centers with different levels of facilities particularly in areas with limited resources.

***Process***

There is no unique approach for diagnosing myocardial contusion, with the only conclusive test being autopsy. Therefore, we propose a suitable approach from the analysis of a number of papers, and we offer a reasonable and affordable guideline based on available statistical data and scientific evidence. A computerized search of MEDLINE was undertaken using PubMed interface. English language citations were enquired for myocardial contusion from 1977 through 2012 using the primary search strategy: (*Myocardial Contusion*) AND (*Blunt Chest Trauma*). Articles were selected from joint research centers of cardiology and cardiovascular surgery. The related articles of PubMed were also selected and used to identify the articles similar to primary strategy. Out of 104 articles recognized by these 2 methods, 33 were confirmed for myocardial contusion after blunt chest trauma. Each article was reviewed by 2 of our authors of the work group. Data were collected, and a consensus was achieved for the final opinion and recommendations vis-à-vis this guideline.

***Questions to be Addressed***

The most important questions in dealing with trauma patients are:

1. What to do with patients suspected of having myocardial contusion?
2. What is the first step in dealing with patients suspected of having a myocardial contusion?
3. What steps follow normal test results and abnormal results?
4. How long of a hospital stay is necessary, and what patients are fit for discharge?
5. Are there any additional measures needed after discharge?
6. Which significant thoracic trauma patients need surgery?

A logistic approach to treating patients with suspected myocardial contusion could be explained by answering a few key questions:

1. Which patients are suspected of having a myocardial contusion injury (MCI) after blunt thoracic trauma (BTT)?^5^^, ^^11^

MCI is an uncommon side effect of BTT and usually results from a motor vehicle accident or an accident caused by sudden loss of speed, which is called a deceleration injury. It can also result from a direct blow to the chest.

The exact incidence of MCI after BTT is unknown, but a study has reported it to be between 8% and 71%.^11^ A definite diagnosis can be achieved only by direct viewing at autopsy. Strong suspicion is required to diagnose MCI. Patients who sustain significant thoracic trauma are susceptible to MCI. In these situations, significant trauma includes multiple rib fractures, hemothorax, pulmonary contusion, and intrathoracic vascular injury. Patients with these injuries have a 13% chance of blunt myocardial contusion. They may also experience pain such as angina and infarct that is not reduced with analgesics. Patients may have shortness of breath; therefore, the examination should be nonspecific. They may have flail chest, chest wall tenderness, dysrhythmias or ectopic beat, or sinus tachycardia. Sometimes they have no common symptoms. It should be noted that a sternum fracture does not necessarily indicate significant damage. Therefore, the patient’s symptoms and a physical examination are not specific for diagnosing MCI. Based on the mechanism of trauma severity, appropriate diagnostic measures should be undertaken. 

2. What is a reasonable and appropriate diagnostic measure for a patient suspected of having MCI? 

Based on trials made on such patients, the class I recommendation is 12-lead ECG. Notably, a right ECG (V4R) adapted on the right ventricle (RV) and an ECG with limited leads are not helpful; consequently, the 12-lead ECG is the first step for a patient suspected of having MCI.^12^^, ^^13^

ECG is a sensitive method and the best single predictor of MCI. Indeed, between 40% and 83% of MCI patients will have an abnormal ECG. Maximum abnormality which will be visible in an ECG is arrhythmia, which is generally seen within the first 24–48 hours. Ventricular fibrillation is the most common cause of death. Left ventricle (LV) contusion will be present as an abnormality of ST-T and pathologic Q waves. Atrial fibrillation is correlated with poor outcomes. A right bundle branch block is common. First-degree atrioventricular (AV) block, left bundle branch block, hemi-blocks, and third-degree AV block are also seen. 

Unfortunately, there is no relation between the severity of contusion and arrhythmia, and the ECG changes associated with the effects of trauma are weak.^6^^, ^^14^

Some studies have shown that measuring troponin levels is useful as screening before an ECG.^15^^-^^18^ In a patient who is suspected of having MCI, one will be able to decide by evaluating troponin levels and an ECG:

1. If the ECG is normal and the troponin test is negative, MCI is almost ruled out, and, if stable, the patient can be discharged.

2. If the initial ECG is abnormal, 24–48 hours of monitoring is recommended. To increase sensitivity, it is better to compare a second ECG with the previous one.

3. If the first ECG is abnormal and the troponin test is positive, the next diagnostic step is required. The most appropriate and cost-effective step is echocardiography (class II recommendations).^5^^, ^^6^

Transesophageal echocardiography (TEE) is more sensitive than is transthoracic echocardiography (TTE) because it is not necessary to maintain the proper position (unlike TTE) but the thoracic aorta is not seen, and 90% of aortic ruptures occur in the isthmus which illustrates the rupture well. The RV outflow tract cannot be seen with TTE, but it can be detected with TEE. TTE does not have a good correlation with ECG and enzyme testing. Echocardiography is reasonable and necessary under the following conditions:

1. When the initial ECG is abnormal and the troponin test is positive.

2. When a trauma patient is in shock and noncardiac causes are rejected.

3. When hemodynamic instability or arrhythmia is seen in the ECG. If TTE is not helpful and there is a suspicion of cardiac causes, TEE is recommended.^5^^, ^^6^^, ^^19^^-^^22^

It is noteworthy that sternum fracture does not mean MCI, and monitoring is not required in the presence of a normal ECG and negative troponin test. If the chest X-ray, ECG, and troponin level are normal, the patient can be discharged. 

In a retrospective study, 67 out of 100 patients had isolated sternum fractures, and based on abnormal ECG results, MCI was reported at 4%.^23^ Therefore, echocardiography is not recommended in isolated sternum fracture.^23^

The probability of these disorders can be seen in echocardiography. Accordingly, subsequent treatment steps can be determined based on the following observable impairments in MCI patients: 

1. Angiography is needed when a wall motion abnormality and reduced ejection fraction is seen in an echocardiogram with an ECG abnormality.

2. When there is significant effusion and tamponade, requiring intervention and surgery.

3. Significant valvular heart disease and LV dysfunction requiring immediate surgery are seen in the echocardiogram 

4. In cases of acute deterioration of cardiac function following trauma, normal angiography treatment with positive inotrope and intra-aortic balloon pump is essential.

5. Ruptured structure will be decided based on the severity of visible abnormalities like ventricular septum, ventricular aneurysm, and aortic fistula to heart chambers.^6^

A small percentage of MCI patients with myocardial infarction symptoms will refer to hospital. Most of them are not actual coronary stenosis and will show ST changes 5–7 days after trauma. These patients definitely require angiography but often have normal coronary activity. The reason for these patients having a myocardial infarction could be in-situ thrombosis or dissection. If they are not barred from taking anticoagulants, percutaneous coronary intervention can be performed.

3. Under what conditions is a nuclear study reasonable?

Myocardial perfusion using TC^99^ is capable of demonstrating a reduction in myocardial perfusion, but it is not recommended for the diagnosis of MCI and is not reasonable for routine use. 

Myocardial perfusion with thallium is useful for left ventricular (LV) evaluation, but because of the right ventricular (RV) position, right ventricular (RV) contusion has doubled the LV.^24^^-^^27^ Some studies have shown that scanning is not useful for examining MCI.^24^^-^^27^

4. Is the study of myocardial metabolism with positron emission tomography (PET) valuable in trauma?^28^^, ^^29^

Metabolism can be evaluated with PET and single-photon emission computerized tomography (SPECT) perfusion, if echocardiography and ECG results are abnormal and coronary angiography is normal. If perfusion and metabolic defects are matched, an infarct diagnosis will be established. The first report of using PET metabolic in a 45-year-old who was suspected of having myocardial infarction was discussed in a study by Pai M. et al.^29^

Today, the best method to assess myocardial viability is PET with fluorine-18 (^18^F-FDG), although it is not recommended for routine use. 

If the evidence is not enough in a person suspected of having MCI, it is in the recommendations of class III, and the following points are useful:

1. Routine troponin checks for all trauma patients and a serial check in the case of abnormality are recommended. (Optimal time of control is not mentioned.)
2. In a patient with normal echocardiography, CT scan or heart magnetic resonance imaging, angiography or drug therapy is not useful.
3. Surgery is reasonable with monitoring and use of a pulmonary artery catheter^30^ (Swan Ganz) in elderly patients with heart disease and unstable conditions associated with an abnormal ECG.

**Figure 1:**
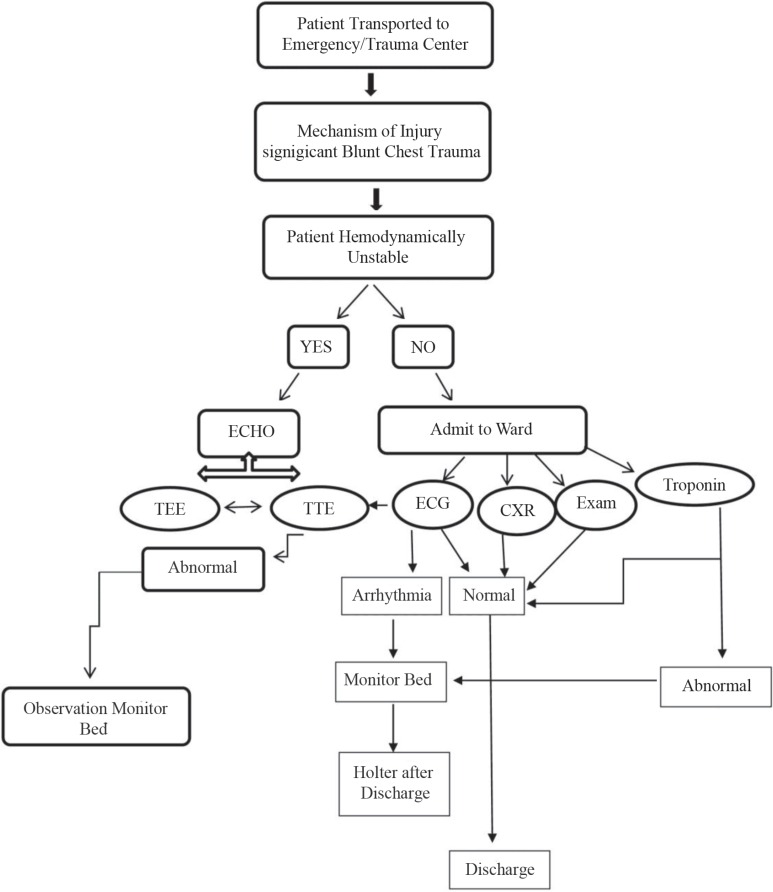
Blunt chest trauma algorithm for the detection of myocardial contusion injury (MCI) in trauma patients.

ECHO, Echocardiography; TEE, Transesophageal echocardiography; TTE, Transthoracic echocardiography; ECG, Electrocardiography; CXR, Chest X-Ray

In short, a new algorithm for the detection of MCI in trauma patients admitted to the trauma emergency ward is shown in Figure 1.

***Cardiac Enzymes***

Cardiac enzymes are small molecules released in blood circulation for whatever reason following damage to the myocardial cell membrane. There are several types of cardiac enzymes and the most specific are troponin I and troponin T. Other enzymes such as keratin phosphokinase and myoglobin have a great deal of false positive with increases of CPK MB in terms of muscle mass trauma and are not recommended for trauma patients.

***Sternal Fractures***

Today, sternal fracture does not reflect the severity of trauma and has no additional diagnostic value with echocardiography and indication of normal troponin enzyme. Therefore, we will be able to safely discharge these trauma patients. 

***Surgical Repair***

In trauma patients, surgical repair is needed in significant and serious valvular damage. If valve damage is leading to heart failure and shock, surgical repair and/or valve replacement is inevitable. In the case of myocardial rupture leading to cardiac tamponade or coronary artery laceration or aorta, surgical repair is reasonable and necessary.

***Other Miscellaneous Diagnostic Tests***

The indication of routine, time-efficient, and cost-effective tests in conditions of suspected contusion injury has been described in the text. Tests such as nuclear cardiology scans and myocardial perfusion scans are too costly. In some patients who experience rib and lung injuries associated with the accident leading to heart contusion, other tools may be used to determine if there are significant injuries to the heart, ribs, or arteries or if there are fractures. These tools include computed tomography (CT) scan of the heart, chest X-ray, and ECG to monitor heart activity, and echocardiogram to visualize the flow of blood through the heart.^31^ The results of a study by Elie et al.^32^ showed that utilizing a combination of echocardiography, ECG, and troponin tests for suitable patients might advance the diagnosis and risk stratification of blunt trauma injury patients.

***Future and Controversy***

The future pathway to improving management and diagnosis of blunt chest trauma involves diagnostic tests, endovascular techniques, and patient selection. The use of thoracoscopy for the management and diagnosis of thoracic injuries will increase. The use of ultrasounds for diagnosis in cases such as hemothorax and cardiac tamponade will become more widespread. Finally, CT scanning techniques will be used more frequently to diagnose major injuries to the thoracic aorta and its branches. Endovascular techniques will be developed further, and they will be used more frequently to repair great vessel injuries. Nonsurgical techniques and patient selection will be redefined for delayed operative management of thoracic aortic rupture.^33^

***Recommendations***

- The first step in the diagnostic and class I recommendations is ECG in all trauma patients with suspected myocardial contusion based on trauma mechanism.
- Control of the cardiac enzyme is not recommended by class I, but control of simultaneous cardiac enzymes together with ECG in patients strongly suspected of having a contusion is a class II recommendation.
- Echocardiography is not the first line of diagnosis. If the echocardiogram is abnormal, cardiac enzymes and shock are unjustified based on surgical causes, and therefore echocardiography is logical and recommended as a component of class II.

## Conclusion

A myocardial contusion refers to a bruise of the cardiac muscle secondary to trauma, the severity of which can vary depending on the severity of the transferred energy to the heart and preexisting heart’s conditions. Diagnosing myocardial contusion is difficult due to the patient’s nonspecific symptoms and the lack of an ideal diagnostic test. It is necessary to have an algorithm about how to approach to the patient when there is a possibility of myocardial contusion with respect to local facilities.

## Notes:

***This paper should be cited as:*** Alborzi Z, Zangouri V, Paydar S, Ghahramani Z, Shafa M, Ziaeian B, Radpey MR, Amirian A, Khodaei S. Diagnosing Myocardial Contusion after Blunt Chest Trauma. J Teh Univ Heart Ctr 2016;11(2):49-54.
